# Five autoantibodies identified from immune complexes as breast cancer biomarkers

**DOI:** 10.3389/fimmu.2025.1640054

**Published:** 2025-07-22

**Authors:** Ningwei Zheng, Yueqi Li, Zhengke Peng, Yaolin Tang, Zhiqiang Liang, Hong Wang, Hong Dai, Gongjun Tan

**Affiliations:** ^1^ Department of Clinical Laboratory, Zhuhai Center for Maternal and Child Healthcare (Zhuhai Women and Children’s Hospital), Zhuhai, China; ^2^ Department of Clinical Laboratory, Zhuhai Hospital, Jinan University, Zhuhai, China; ^3^ Department of Breast Surgery, Zhuhai Center for Maternal and Child Healthcare (Zhuhai Women and Children’s Hospital), Zhuhai, China; ^4^ Department of Gynecology, Zhuhai Center for Maternal and Child Healthcare (Zhuhai Women and Children’s Hospital), Zhuhai, China

**Keywords:** complement 1Q, immune complex analysis, autoantibodies, breast cancer, digital liquid chip method

## Abstract

**Objective:**

Comprehensive identification and profiling of antigens in serum immune complexes (ICs) is crucial for developing early diagnostic biomarkers for cancer. We therefore undertook this study to identify novel IC-derived autoantigens and autoantibodies in patients with breast cancer, and to evaluate their potential as new biomarkers.

**Methods:**

ICs were purified from serum with C1q and Protein A/G affinity capture. The isolated complexes were digested with papain and analyzed by liquid chromatography-tandem mass spectrometry (LC-MS/MS). Twelve candidate autoantibodies revealed by LC-MS/MS were first verified with a digital liquid chip method (DLCM) in baseline serum from 40 breast cancer patients and eight healthy controls. Five autoantibodies were then validated in independent cohorts of 33 breast cancer patients and 45 healthy controls, using DLCM.

**Results:**

Autoantibodies targeting PF4, PSMB3, PRPF19, RTCB, SDHA, ENO1, PTBP2, PRDX6, ANP32A, VDAC1, MMP14 and HSPA4 were identified both purification methods. In the verification cohort, IgG autoantibodies against HSPA4, ENO1, PRDX6, PRPF19 and MMP14 were significantly increased in breast cancer patients with areas under the curve (AUCs) of 0.90, 0.89, 0.82, 0.78 and 0.77, respectively. Their combined panel discriminated breast cancer from controls with an AUC of 0.97. In the validation cohort, the same autoantibodies achieved AUCs of 0.79, 0.81, 0.73, 0.87, and 0.82, and the combination of these five autoantibodies yielded an AUC of 0.88.

**Conclusions:**

The autoantibodies identified from ICs can serve as effective serum biomarkers for breast cancer. Anti-HSPA4, anti-PRPF19, anti-ENO1, anti-PRDX6, and anti-MMP14 autoantibodies showed significant increases in breast cancer patients.

## Introduction

Although evidence that the immune system can restrain tumors dates back more than a century, cancer immunology is still considered a burgeoning field (1). Groundbreaking discoveries have recently clarified how distinct immune components surveil emerging tumors or, conversely, foster their progression, underlining their roles in tumorigenesis and their prognostic value (2, 3). Both innate and adaptive immune cells now appear to influence cancer development directly or indirectly, even at pre-cancerous stages (4). Tumorigenesis gives rise to abnormal, antigenic molecules that can trigger immune responses and the production of tumor-associated autoantibodies (5, 6). These tumor-associated antigens (TAAs) remain the biomarkers most commonly measured and the targets most often pursued in cancer management and immunotherapy (7). However, because circulating TAAs concentrations usually rise in proportion to tumor burden, their usefulness for early diagnosis is limited (8). Autoantibodies have been detected for a range of cancers at an early stage before development of clinical symptoms. Their detections are minimally invasive, cost-effective, and easily performed with established technologies (6). Moreover, antibodies circulate with half-lives of up to roughly 30 days and are more stable ex vivo than many other biomarker types, making them attractive tools for early cancer detection and prognosis.

Based on these observations, identification of the autoantigens that are recognized by autoantibodies is important for understanding antigen recognition in both B cell and T cell immunity. Indeed, to date, a great diversity of tumor autoantigens has been identified in different tumors by using autoantibody discovery technologies, such as protein chip (6), serological analysis of recombinant cDNA expression libraries (SEREX) (9) and phage display (10). However, these autoantigens have not been directly shown to form immune complexes *in vivo* in cancer patients, which form because of an immune response. Current discovery methods rely on *in vitro* reactions between alternative antigens, which are recombinant proteins and do not reflect protein changes leading to *in vivo* immunogenicity, and antibodies come from serum of patients (11).

Immune complexes (ICs) are produced by the union of one or more antibody molecules with one or more antigen molecules (12). It is reported that ICs in breast cancer not only determine clinical pathologic staging, but also seem to be helpful in prognostic assessment because they distinguish between patients who were free of disease and those who had relapsed or died (13). Lastwika et al. (14) found most of the tumor-derived autoantibodies were present in plasma as both free and complexed to an antigen. To address this gap, we developed a method that combines immune-complex purification with complement component C1q, which specifically binds ICs, thereby enabling comprehensive profiling of IC-associated antigens (15, 16). We previously validated this approach in sera from patients with systemic lupus erythematosus (17). In this study, we applied our immune-complex analysis to breast cancer sera to identify IC-associated autoantigens and to evaluate the corresponding autoantibodies as potential biomarkers of the disease.

## Methods

Serum samples were obtained from 73 newly diagnosed breast cancer patients (aged 34–60 years) at Zhuhai Maternity and Child Healthcare Hospital. Concomitantly, 53 healthy controls (aged 30–55 years) were enrolled to serve as the reference group. Details are provided in the **Supplementary Materials**. The verification cohort included 40 cancer patients and 8 healthy controls, comprising:

- 5 patients with imaging-based BI-RADS staging;
- Pathological TNM staging: 5 cases of carcinoma *in situ* (stage 0), 14 stage I, 12 stage II, and 4 stage III.

The validation cohort included 33 cancer patients and 45 healthy controls, comprising:

- 6 patients undergoing BI-RADS staging;
- Pathological TNM staging: 2 cases of carcinoma *in situ* (stage 0), 9 stage I, 12 stage II, and 4 stage III.

All experimental protocols were approved by the Institutional Ethics Committee of Zhuhai Center for Maternal and Child Healthcare, in strict adherence to the Helsinki Declaration.

Immune complexes (ICs) were purified using magnetic beads conjugated with Protein A/G (PureProteomeVR, Millipore, Darmstadt, Germany) and micro-plates immobilized with C1q (DRG Instruments GmbH, Germany). The purified ICs underwent sequential processing: papain digestion, in-solution tryptic digestion, and analysis by nano-liquid chromatography-tandem mass spectrometry (nano-LC-MS/MS). Autoantibodies were detected using the Digital Liquid Chip Method (DLCM).

### Isolation of ICs from pooled serum

**Figure 1:**
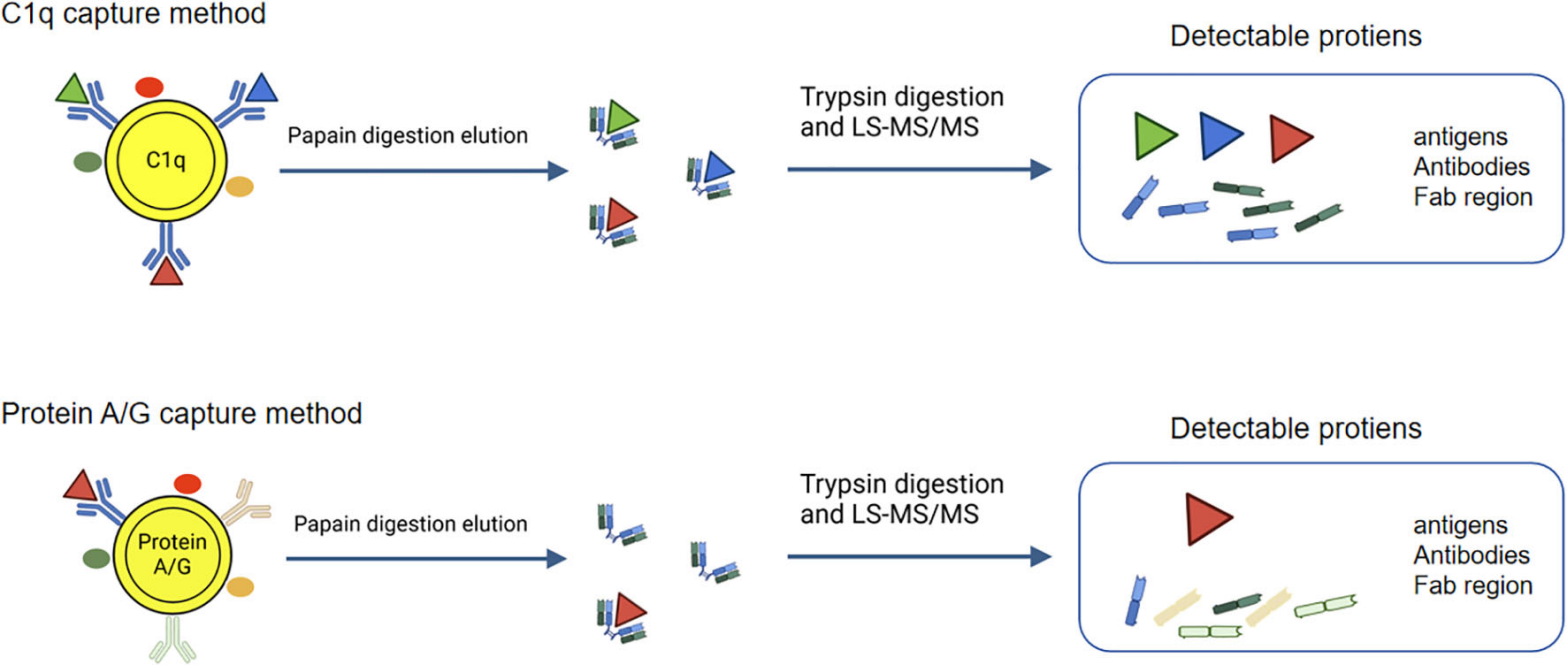
ICs purified by two protocols. C1q capture method: The solid-phase carrier is coated with C1q to capture ICs in serum. The Fab fragments are released by Papain digestion, followed by tryptic digestion and identification via LC-MS/MS. Protein A/G capture method: The liquid-phase carrier contains Protein A/G magnetic beads to adsorb ICs in serum. The Fab fragments are released by Papain digestion, followed by tryptic digestion and identification via LC-MS/MS.

Two methods—C1q-based capture and Protein A/G-based capture—were used to extract immune complexes (**Figure 1**).

#### ICs were purified using immobilized protein A/G magnetic beads (HY-K0202–5 mL, MEC^®^)

Specifically, 10 μL of serum was diluted to a final volume of 100 μL with phosphate-buffered saline (PBS). The diluted serum was gently vortexed and incubated with the immobilized beads for 30 minutes at room temperature. Following magnetic separation for 1 minute, the supernatant was carefully aspirated. The beads were then resuspended in 500 μL of PBS, subjected to magnetic separation for 1 minute, and the supernatant was removed. This washing procedure was repeated three times to ensure thorough purification (15).

#### ICs purified by microplate with immobilized C1q

Microplate with immobilized C1q was incubated with 10 μL of human pooled serum diluted with 500 μL PBS for 30 min with gentle mixing, then the supernatant was removed, and followed by 3 times of wash using 500 μL of PBS (15).

#### Papain-digestion of IC-antigens

50 μL of papain solution (0.04M EDTA, 0.04M L-cysteine) was added to the microplate or the tube containing beads and incubated at 37°C for 30 min respectively. The digested supernatant was then transferred to a new tube, and 50 μL of 0.06M iodoacetamide was added to terminate the papain digestion (15).

### Identification of supernatant proteins by nano-LC-MS/MS

#### Enzymatic hydrolysis of sample

After reduction and alkylation, Trypsin (mass ratio 1:50) was added and enzymolyzed at 37°C for 20 hours. The enzymatic hydrolysate was desalted, lyophilized, dissolved in 0.1% formic acid (FA) solution, and stored at -20°C for subsequent use.

#### Mass spectrometric analysis

Liquid A: an aqueous solution of 0.1% FA, and liquid B: an aqueous solution of acetonitrile of 0.1% FA (acetonitrile occupies 84%). After the chromatographic column was balanced with 95% liquid A, the sample was loaded from the automatic sampler to the Chrom-Trap.

#### Mass spectral data collection

The mass charge ratio of polypeptides and polypeptide fragments was collected by the following method: 20 fragment maps (MS2 scan) were collected after each full scan.

### Data analysis

The raw mass spectrometry file was retrieved from the corresponding database using Proteome Discoverer 1.4 software to obtain the identified protein results. The parameters for database searching were as follows:

- Enzyme: Trypsin
- Database: uniprot_Homo_sapiens_194324_20210106
- Fixed modifications: Carbamidomethyl (C)
- Variable modifications: Oxidation (M)
- Missed Cleavage: 2
- Peptide Mass Tolerance: 20 ppm
- Fragment Mass Tolerance: 0.1 Da
- Filter by score >=20

### Verification and validation of serum biomarkers using digital liquid chip method

First, the recombinant proteins (Sangon Biotech, China; see **Supplementary Material** for specific catalog numbers) were coated onto barcoded magnetic beads (BMBs) and reacted with serum. The mixture was incubated on a shaking bed at 37°C for 15 min, followed by elution and aspiration. Subsequently, 50 μL of P-phycoerythrin (PE)-labeled secondary antibody was added to each well. After another 15 min incubation on the shaking bed at 37°C, the wells were eluted and aspirated again. Finally, 100 μL of enhancement solution was added to each well, and the magnetic barcode fluorescence intensity (MFI) was measured at λ=530nm.

## Results

**Figure 2:**
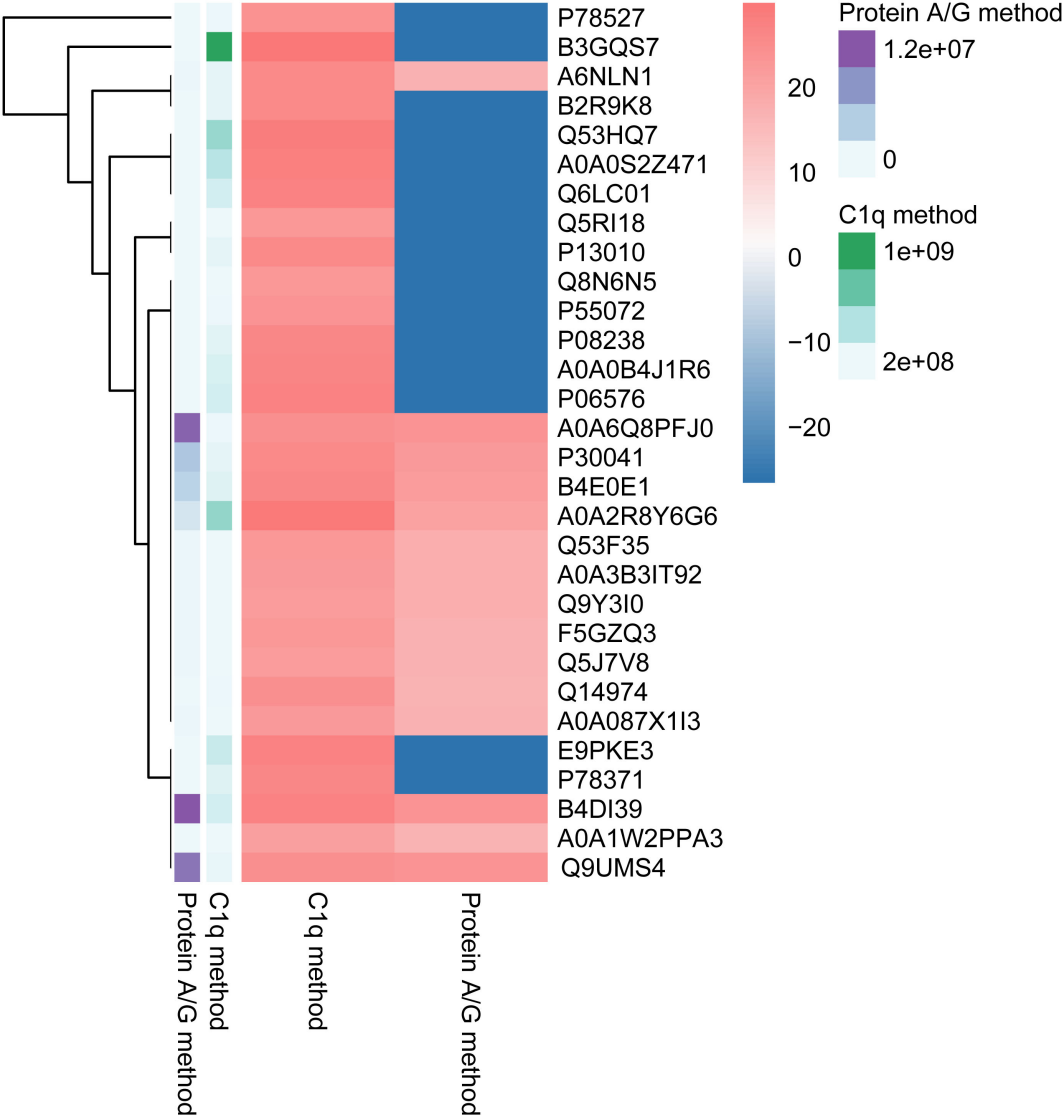
Comparison of the number of human proteins identified by two methods (top 30 proteins). The horizontal axis represents two detection methods: the C1q method and the Protein A/G method. The vertical axis lists various protein identifiers. The color gradient reflects the differences in data levels.

We compared ICs in human serum identified with the C1q-capture method versus the Protein A/G method. Five pooled serum samples were processed by each technique and each pool was analyzed in triplicate. The antigen lists obtained from the two methods were then intersected. The C1q method detected 246 distinct IC-associated antigens, whereas the Protein A/G method detected 152 (**Figure 2**); 125 antigens were common to both approaches.

**Figure 3:**
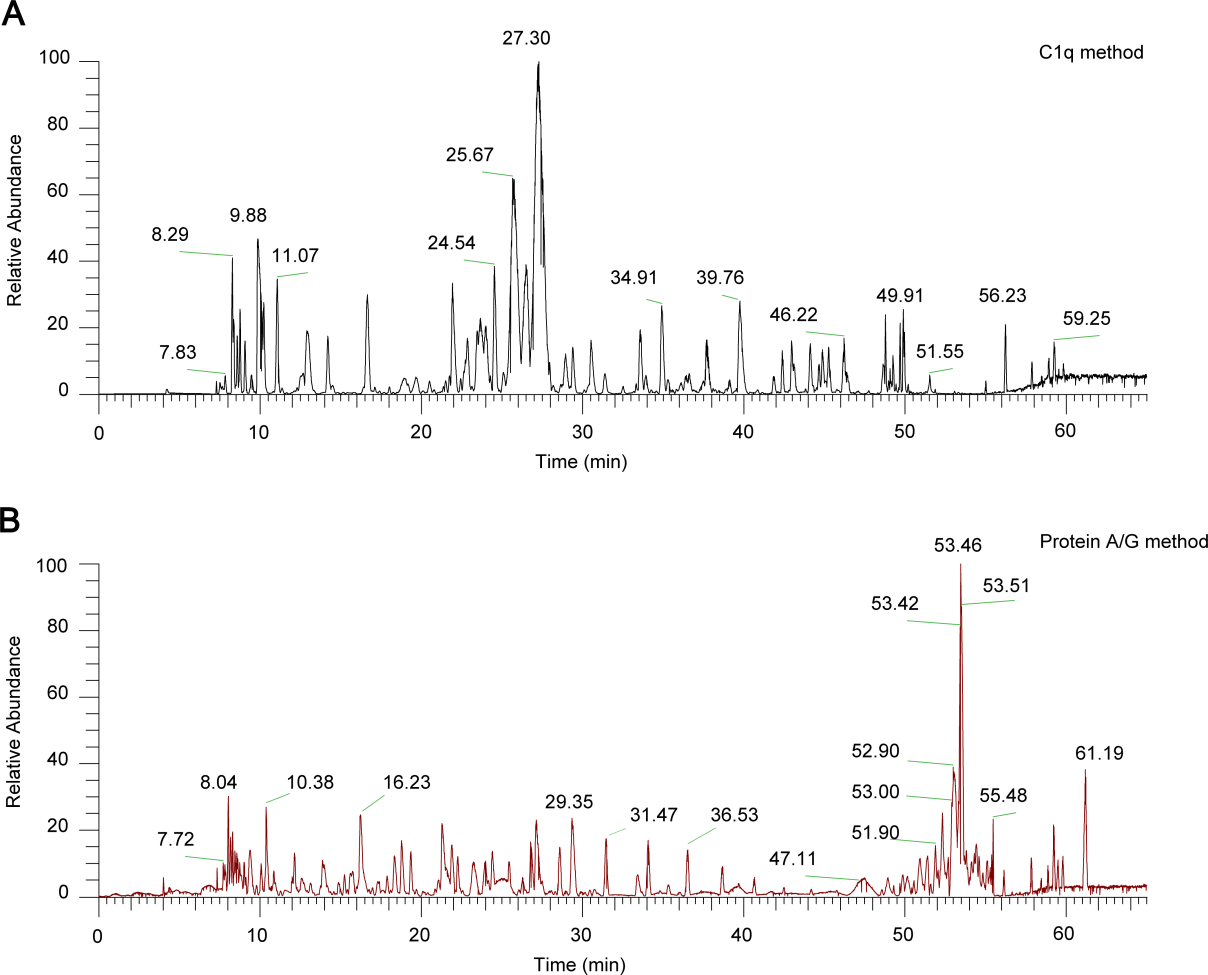
Chromatograms of the two protocols. Chart **(A)** shows a C1q method and Chart **(B)** shows a Protein A/Gmethod.The horizontal axis represents retention time (minutes), and the vertical axis represents relative abundance (%). The number labeled on each peak indicates the retention time (minutes).

The nano-LC–MS/MS chromatograms generated by the two capture methods differed markedly (**Figure 3**). The C1q approach produced a prominent peak at roughly 27 min, whereas the Protein A/G method showed its main peak at about 53 min. In addition, the Protein A/G chromatogram contained substantially fewer peaks overall than the C1q chromatogram.

### Selected antigens of breast cancer

In total, immune-complex analysis uncovered 273 distinct human antigens in breast cancer serum. Of these, 121 were unique to the C1q-capture method. From the 125 antigens detected by both methods, 12 (**Table 1**) were selected for verification and validation as potential breast-cancer biomarkers.

**Table 1:** Selected antigens of breast cancer identified by two methods.

Accession	Description	Abundances (Grouped) C1q	Abundances (Grouped) ProteinA/G
P30041	PRDX6	56515068.54	4570829.316
Q86VV6	MMP14	21164082.75	11240354.35
Q53F35	ANP32A	8123570.511	231105.6818
A0A087X1I3	SDHA	5519583.76	147655.4596
P21796	VDAC1	28199429.42	267207.5359
Q9UMS4	PRPF19	26639147.32	10112658.56
P49720	PSMB3	8167791.211	297100.3572
Q9Y3I0	RTCB	3269975.122	195750.8631
P02776	PF4	2730792.295	6790417.962
A6NLN1	PTBP1	51419532.77	165317.9859
A0A2R8Y6G6	ENO1	492848973.8	1782607.405
B4DI39	HSPA4	149936002.7	12215007.82

PRDX6, Peroxiredoxin-6; MMP14, Matrix metallopeptidase 14; ANP32A, Acidic (Leucine-rich) nuclear phosphoprotein 32 family; member B variant; SDHA, Flavoprotein subunit of complex I; VDAC1, Voltage-dependent anion-selective channel protein 1; PRPF19, Pre-mRNA-processing factor 19; PSMB3, Proteasome subunit beta type-3; RTCB, RNA-splicing ligase RtcB homolog; PF4, Platelet factor 4; PTBP1, Polypyrimidine tract-binding protein 1; ENO1, 2-phospho-D-glycerate hydro-lyase; HSPA4, Heat shock 70 kDa protein 1.

### Performance of the autoantibodies in the verification cohort

**Figure 4:**
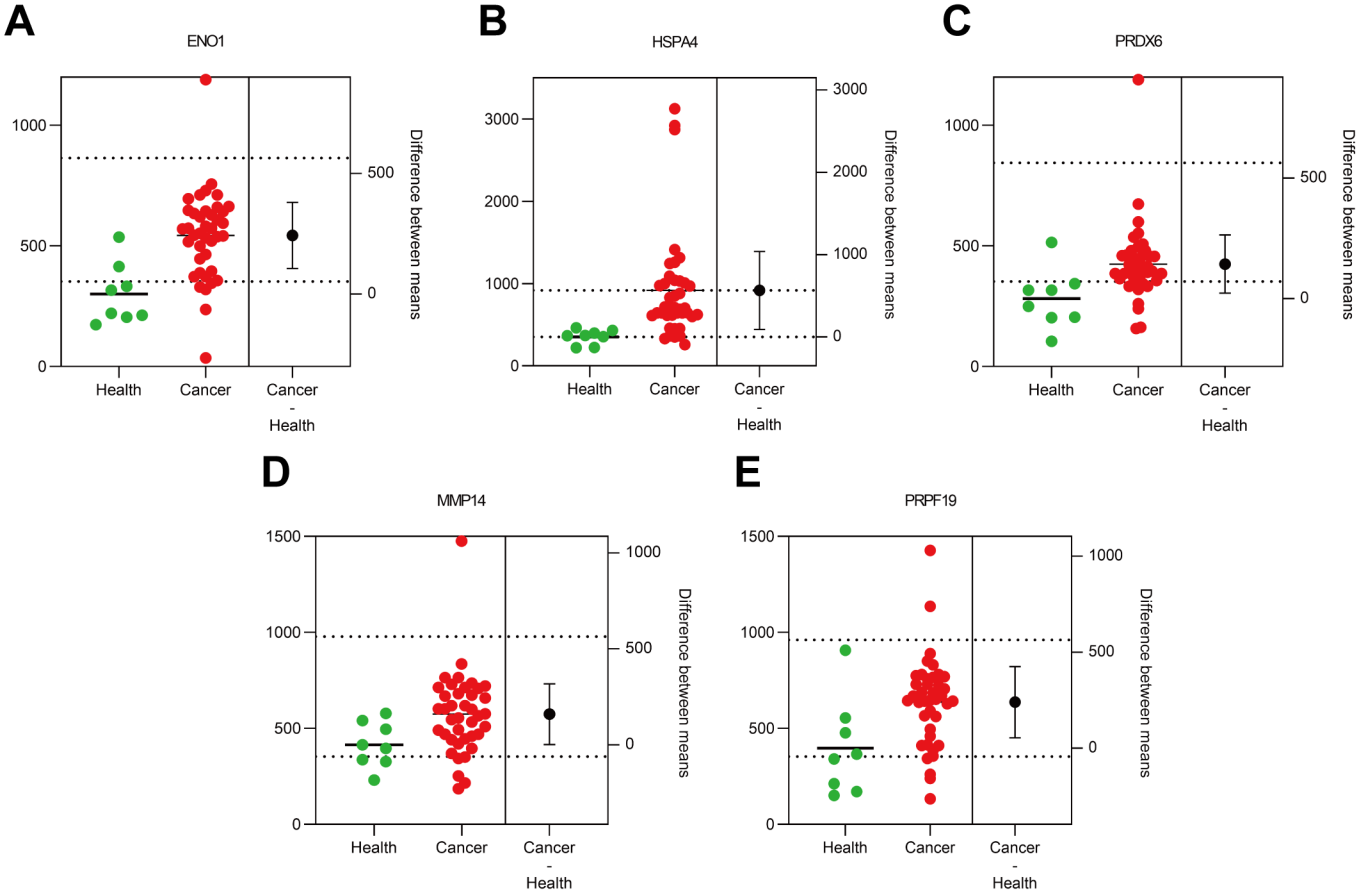
Scatter plot of 5 autoantibodies in the Verification cohort. Scatter plots labeled **(A–E)** compare protein expression levels in health versus cancer conditions.The green dots represent the health group (n=8), and the red dots represent the cancer group (n=40). The black dots denote the difference between means (cancer - health).

**Figure 5:**
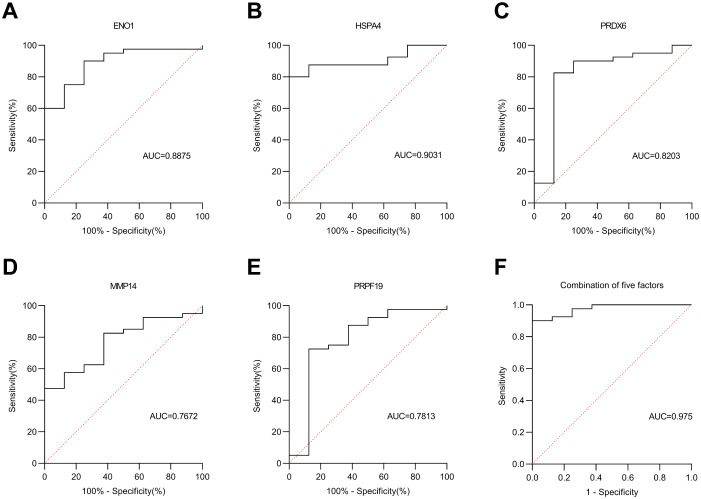
ROC curve analysis in the Verification cohort. **(A–E)** represent the ROC curves of five autoantibodies: ENO1, HSPA4, PRDX6, MMP14 and PRPF19, respectively. **(F)** denotes the ROC curve of the combined five autoantibodies. Solid line: autoantibodies; dashed line: reference line.

In the verification cohort, DLCM quantified 12 autoantibodies (AAbs) in serum from 40 breast cancer patients and 8 healthy controls (**Table 2**). The Mann-Whitney U test showed that five AAbs (HSPA4, PRPF19, ENO1, PRDX6, and MMP14) were significantly higher in the breast cancer group than in the control group (**Figure 4**). The predictive performance of the five AAbs was assessed by receiver operating characteristic (ROC) analysis. Their individual areas under the curve (AUCs) were 0.90, 0.89, 0.82, 0.78, and 0.77, respectively. Combined, these autoantibodies identified breast cancer with an AUC of 0.97 (**Figure 5**).

**Table 2:** Verification assay for specific autoantibodies.

Protein	Healthy		Breast cancer		t	P value
	Mean	SD	Mean	SD		
PF4	2839	626.3	3225	524.6	1.84	0.072
PSMB3	133.5	105.3	179.1	80.98	1.382	0.174
PRPF19	397.1	251.2	636.8	235.5	2.6	0.013
RTCB	396	102.7	731.2	868.9	1.08	0.286
SDHA	137.8	110.7	220.8	186.4	1.211	0.232
ENO1	300.9	125	543.2	183.5	3.557	<0.001
PTBP2	243	119.2	265	104.1	0.533	0.597
PRDX6	281	122.5	425	159.7	2.404	0.020
ANP32A	163.8	89.58	208.4	93.53	1.240	0.221
VDAC1	396.5	185.7	499.9	232.2	1.183	0.243
MMP14	414.9	117.8	573.9	214.7	2.022	0.049
HSPA4	352.6	88.52	916	657.5	2.399	0.021

Healthy (n=8); Breast cancer (n=40). The difference is significant (p<0.05), include HSPA4, MMP14, PRDX6, ENO1 and PRPF19.

### Performance of the autoantibodies in the validation cohort

**Figure 6:**
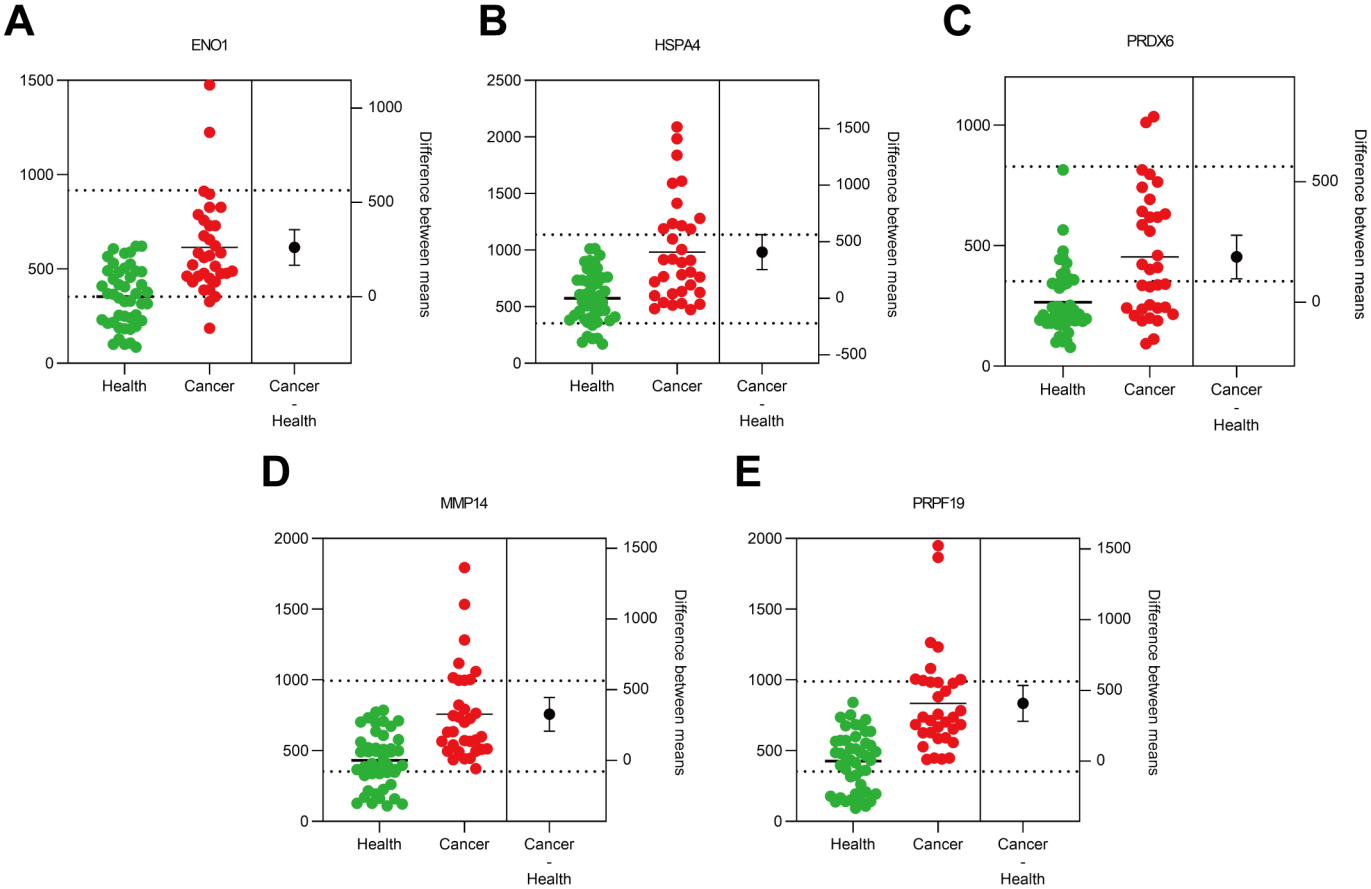
Scatter plot of 5 autoantibodies in the Validation cohort. The green dots represent the health group (n=45), and the red dots represent the cancer group (n=33). The black dots denote the difference between means (cancer - health).

**Figure 7:**
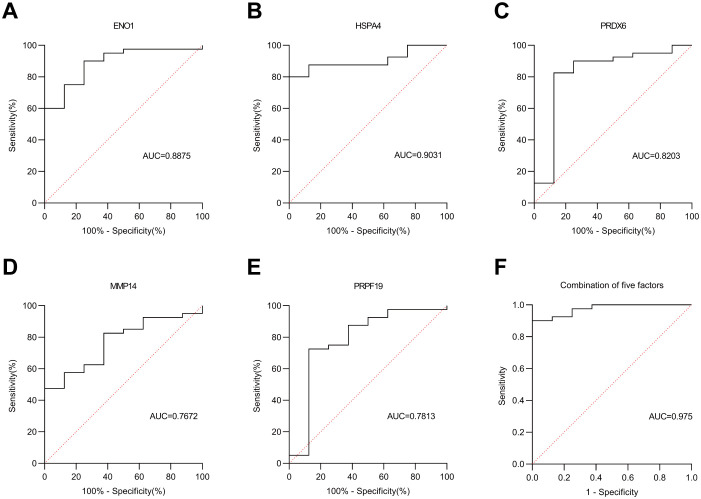
ROC curve analysis in the Validation cohort. **(A–E)** represent the ROC curves of five autoantibodies: ENO1, HSPA4, PRDX6, MMP14 and PRPF19, respectively. **(F)** denotes the ROC curve of the combined five autoantibodies. Solid line: autoantibodies; dashed line: reference line.

We validated these five autoantibodies (HSPA4, ENO1, PRDX6, PRPF19, and MMP14) in an independent cohort of 33 breast cancer patients and 45 healthy controls. Their individual AUCs were 0.79, 0.81, 0.73, 0.87, and 0.82, respectively. Combined, the five-antibody panel detected breast cancer with an AUC of 0.88 (**Figures 6**, **7**).

## Discussion

Antibodies and T cells designed to recognize tumors are now widely used for passive cancer immunotherapy. The relationship between immune complexes (ICs) and cancer has been known for many years (18, 19), but a systematic overview of ICs’ functions in tumors is still missing. Evidence that elevated levels of ICs are closely related to the malignancy of the tumor dates back to the 1970s (19). However, the study of ICs has not captured the attention of clinicians and scientists until the development of mass spectrometry. Identifying specific antigens in ICs in samples from patients with a variety of diseases is not only an important step for developing biomarkers for these diseases but also a potential means to provide information regarding the pathways that contribute to disease pathology. However, a major obstacle to these efforts is the abundant serum proteins. Protein A and Protein G are popular ligands for purifying immunoglobulins (20, 21) and have helped identify IC antigens in autoimmune, infectious, and neoplastic diseases (22). Another option is the C1q-binding assay, which uses the natural affinity of complement component C1q for ICs (22). In our study, based both on the method of “immune complex analysis” and C1q-binding property characterizing immune complexes, we replaced Protein A/G with C1q for immune complex isolation (C1q method, Protein A/G method, as seen in **Figure 1**), aiming to improve both selectivity and sensitivity in the identification of IC-antigens from serum. Results showed a statistically significant difference in the number of identified antigens between the two methods (**Figure 2**). Comparing the chromatograms obtained by nano-LC-MS/MS analysis using each method, many peaks observed using the C1q method disappeared in the chromatograms obtained using the Protein A/G Method (**Figure 3**). We found that C1q method can separate ICs more selectively and sensitively than the Protein A/G method.

We have verified the antibodies in the identified partial autoantibodies (**Figure 3**). Five potential AAb biomarkers (HSPA4, ENO1, PRDX6, PRPF19, MMP14) predictive of breast cancer were identified through verification and validation cohorts (**Figures 4**-**7**). Among these autoantibodies, high serum anti-HSPA4 IgG was correlated with high tumor HSPA4 expression and poor prognosis in breast cancer subjects (23). α-Enolase 1 (ENO1) is a critical glycolytic enzyme whose aberrant expression drives the pathogenesis of various cancers (24). PRDX6 expression is associated with poor prognosis in cancers of multiple tissue origins (25). PRPF19 was positively correlated with liver metastasis and predicted a worse clinical outcome in CRC (26). MMP14 is abundantly expressed on the tumor cell surface (27). The expression levels of five proteins in the CPTAC public database (Data Release: 4.12, June 26, 2025) in tumors and adjacent tissues were analyzed by unpaired t-tests. HSPA4 and PRPF19 were more common in tumors, while ENO1 and PRDX6 were more common in adjacent tissues. Please see the **Supplementary Files**. These proteins are all abnormally overexpressed expressed in tumors or tumor-adjacent tissues of the tumor, thereby triggering the immune system of the organism to initiate responses and generate antibodies against these proteins. Since antibodies are produced in the body earlier than the clinical manifestations of the disease and they are stable in the blood and easy to detect, identifying tumor-specific autoantibodies holds significant scientific and clinical value.

Notably, 8 of the 73 cancer patients in our cohort were at TNM Stage III. After excluding these cases, we performed an unpaired t-test using 65 early-stage cancer patients and 53 healthy controls. Statistical analysis confirmed that the five autoantibodies remained significantly elevated compared to healthy controls (all P < 0.001, unpaired t-test), as detailed in the **Supplementary Materials**. These findings highlight the five autoantibodies as promising candidates for early-stage breast cancer diagnosis.

There are several limitations in this study. Due to the smaller hospital size and short collection time, only a limited number of breast cancer serum samples were available. The diagnostic performance of the AAbs in combination with other biomarkers should also be explored. In addition, the control group should include patients with benign breast diseases and other gynecological tumors to make the evaluation more rigorous and clinically relevant. Finally, in-depth verification of the remaining autoantibodies we identified is needed to obtain more comprehensive information.

## Conclusion

In this study, we identified antigens in ICs from the biological fluids of patients, a strategy that may accelerate the development of diagnostic biomarkers, and ultimately lead to more targeted treatments for breast cancer. Using this method, we comprehensively identified and verified breast-cancer-associated autoantibodies and conducted exploratory biomarker research. This finding holds significant clinical value and promise for future application.

## Data Availability

The original contributions presented in the study are included in the article/**Supplementary Material**. Further inquiries can be directed to the corresponding authors.

## Glossary

ICs: Immune complexes
C1q: Complement 1q
SEREX: Serological analysis of recombinant cDNA expression libraries
DLCM: Digital liquid chip method
Nano-LC-MS/MS: nano-liquid chromatography-tandem mass spectrometry
PBS: Phosphate-buffered saline
Protein A/G: Protein A and protein G
PRDX6: Peroxiredoxin-6
MMP14: Matrix metallopeptidase 14
ANP32A: Acidic (Leucine-rich) nuclear phosphoprotein 32 family
SDHA: Flavoprotein subunit of complex I
VDAC1: Voltage-dependent anion-selective channel protein 1
PRPF19: Pre-mRNA-processing factor 19
PSMB3: Proteasome subunit beta type-3
RTCB: RNA-splicing ligase RtcB homolog
PF4: Platelet factor 4
PTBP1: Polypyrimidine tract-binding protein 1
ENO1: 2-phospho-D-glycerate hydro-lyase
HSPA4: Heat shock 70 kDa protein 1
AUC: Area under the curve
FA: Formic acid
BMB: Barcoded magnetic beads
MFI: Magnetic barcode fluorescence intensity
AAbs: Autoantibodies
ROC: Receiver operating characteristic
