# Supplementary figures and images for: Five autoantibodies identified from immune complexes as breast cancer biomarkers
Source: Front Immunol. 2025 Jul 22;16:1640054. doi: 10.3389/fimmu.2025.1640054 (PMC12324166; doi:10.3389/fimmu.2025.1640054)

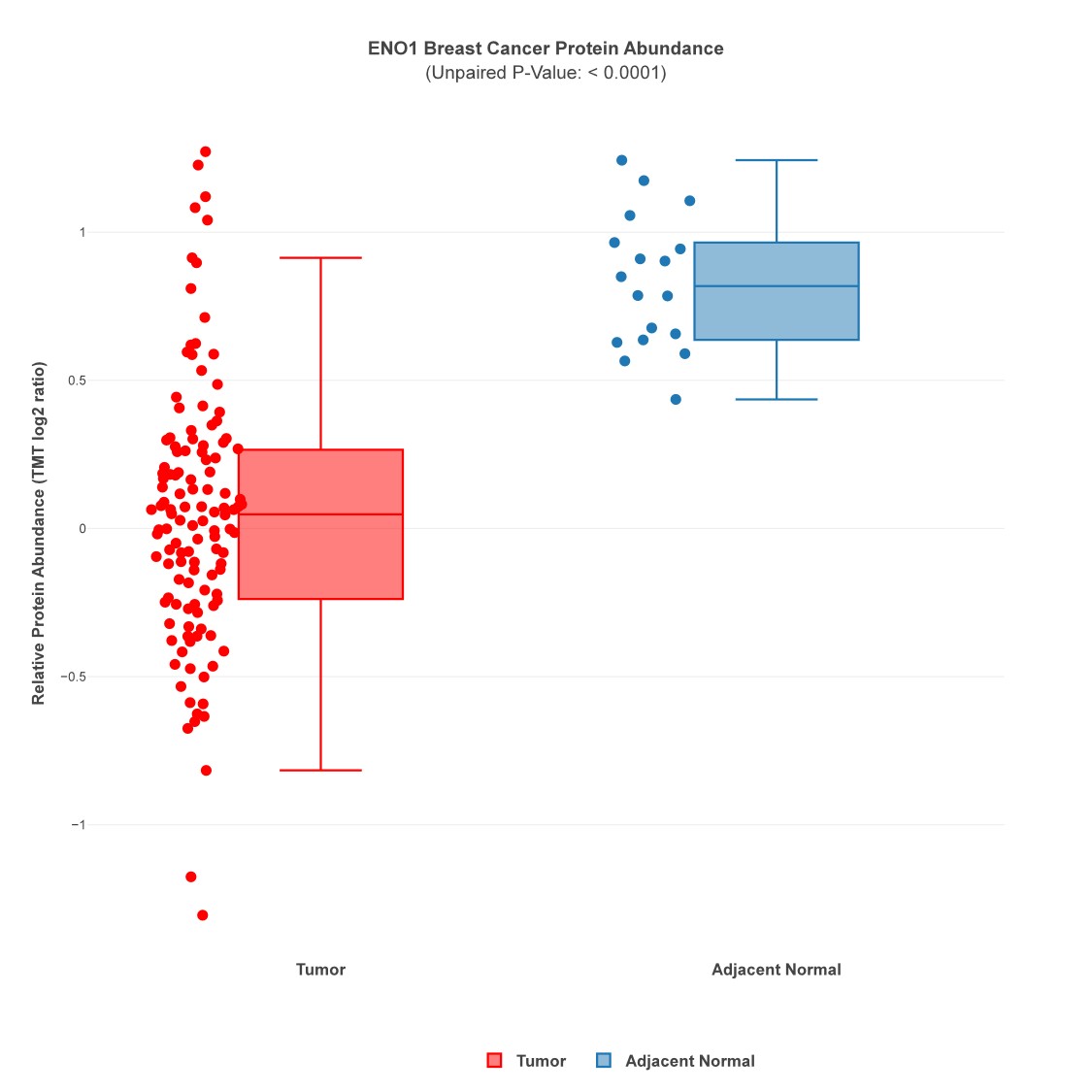

Supplement: Supplementary file 1 [file Image1.jpeg]

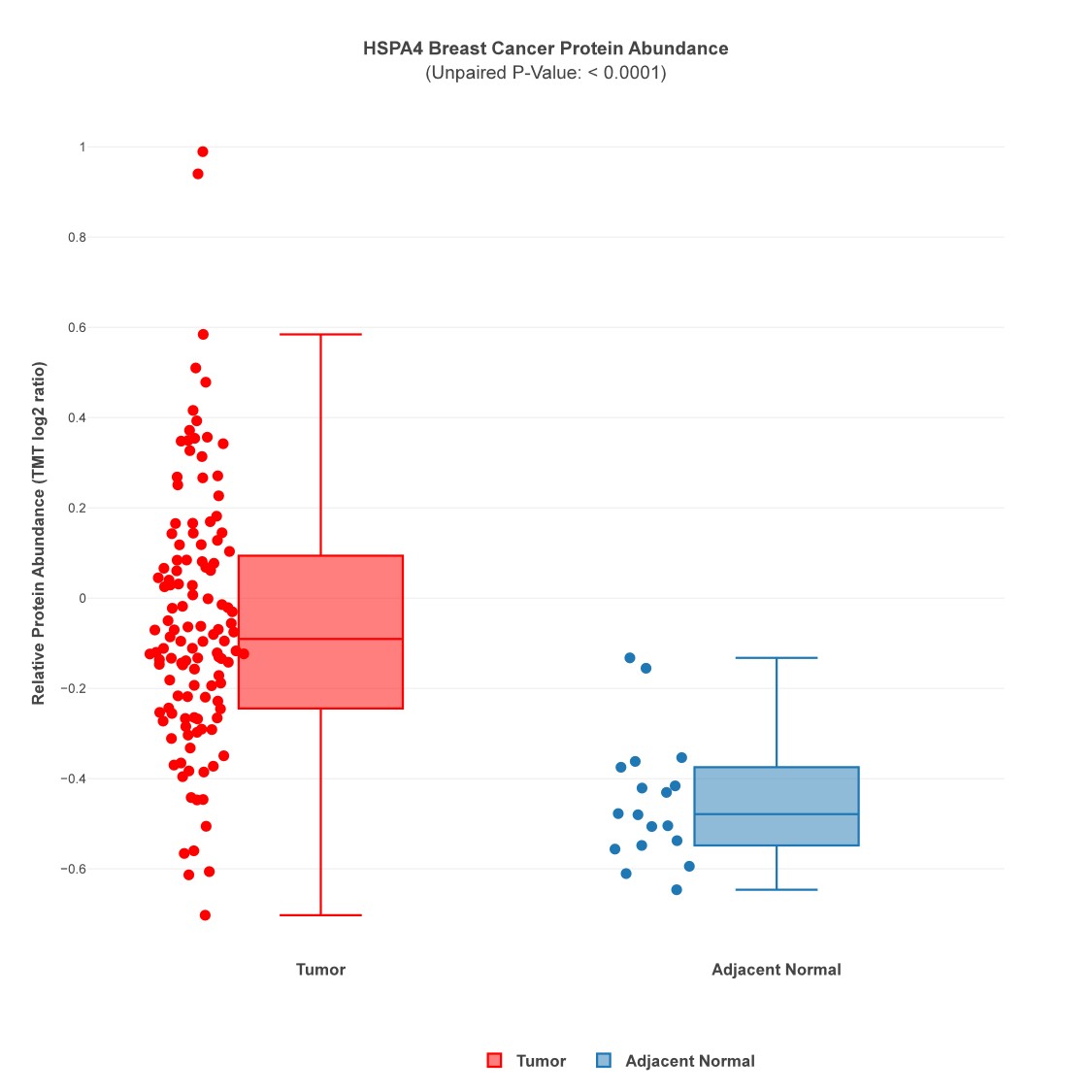

Supplement: Supplementary file 2 [file Image2.jpeg]

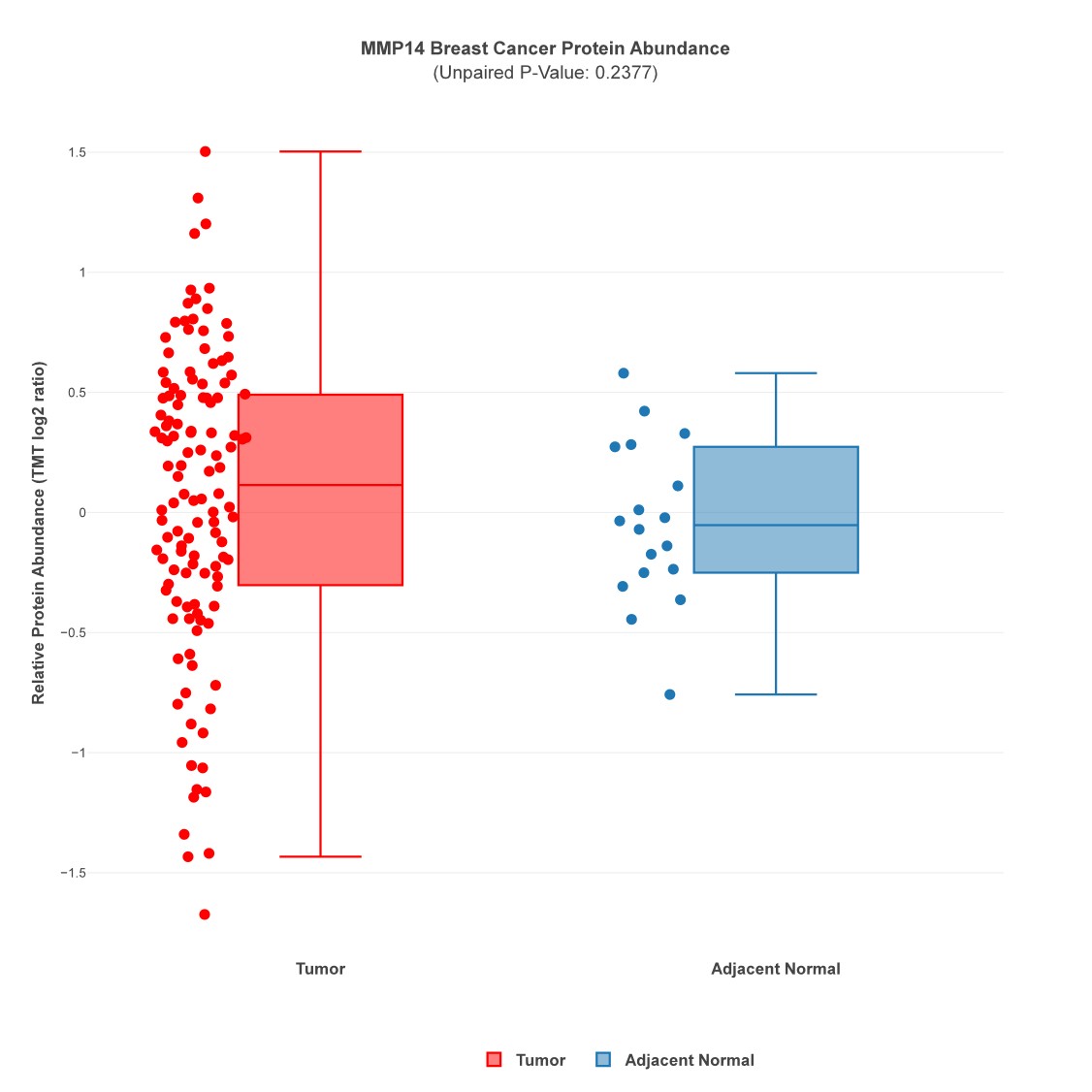

Supplement: Supplementary file 3 [file Image3.jpeg]

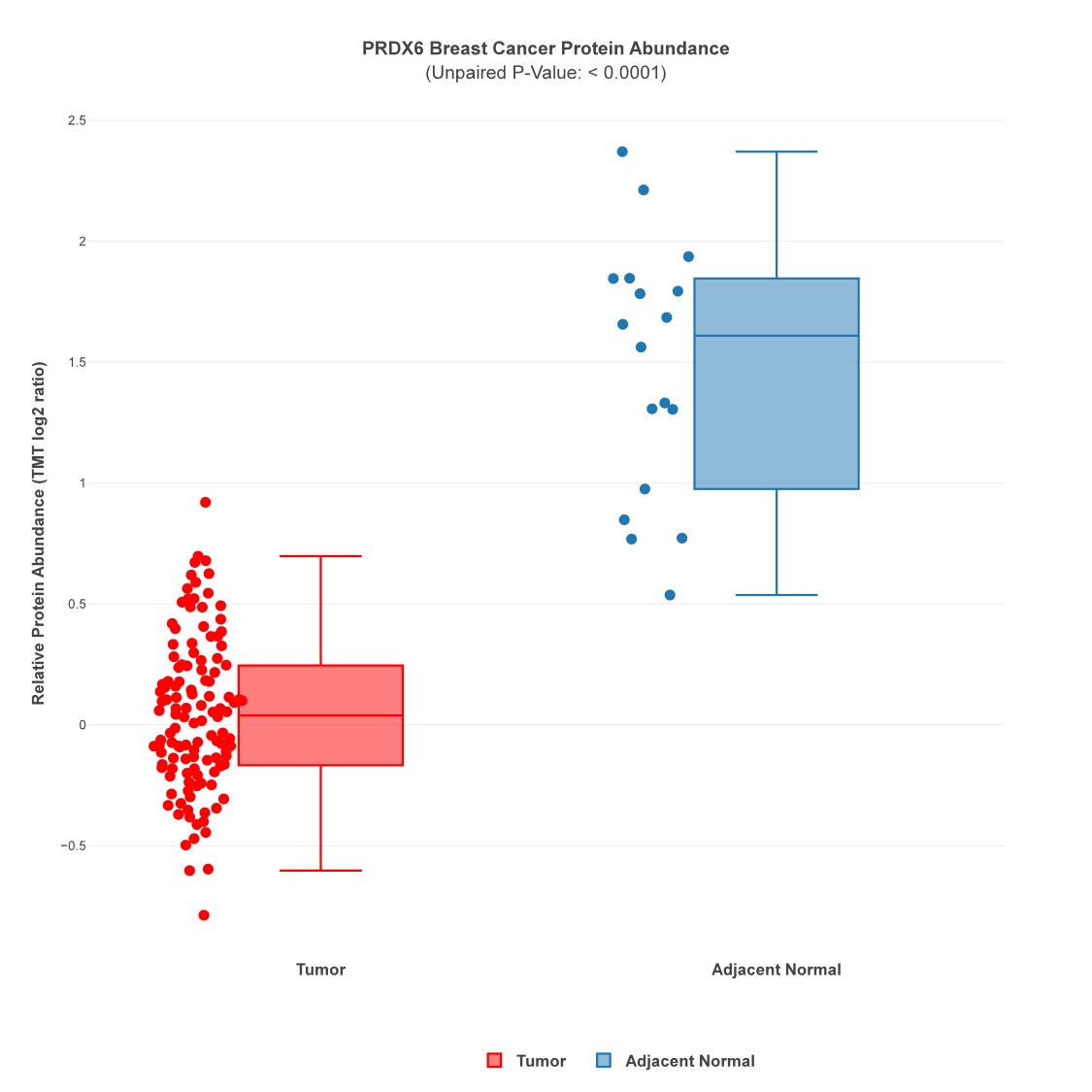

Supplement: Supplementary file 4 [file Image4.jpeg]

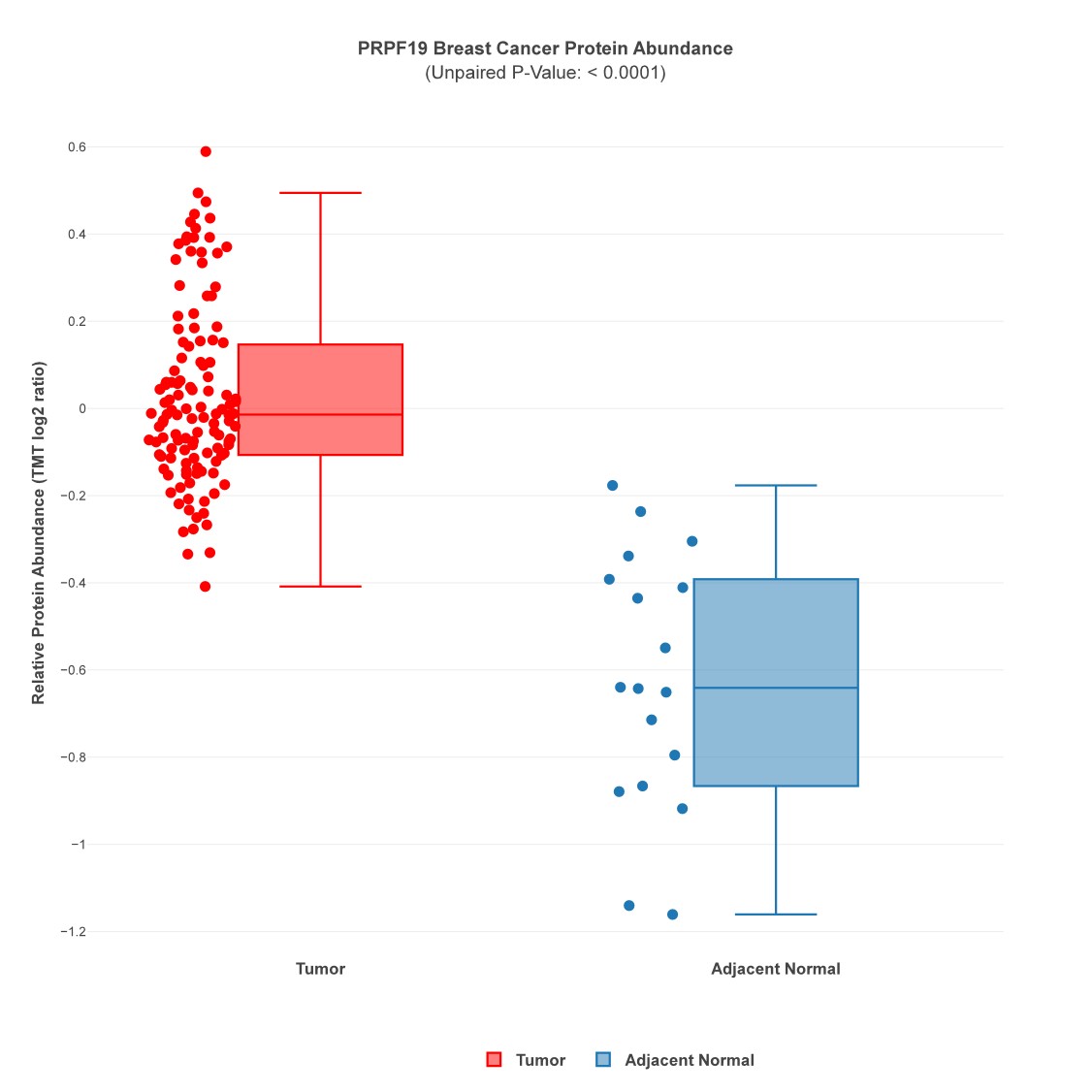

Supplement: Supplementary file 5 [file Image5.jpeg]
